# Oral sirolimus therapy for patients with complex low-flow vascular malformations

**DOI:** 10.1016/j.jvsv.2025.102261

**Published:** 2025-05-19

**Authors:** Rebecca Nisbet, Calver Pang, Nicholas Evans, Ahmed Belhadj, Mohamed Khalifa, Anthie Papadopoulou, Tejinder Randhawa, Jocelyn Brookes, Chung Sim Lim

**Affiliations:** a Royal Free London Vascular Anomalies Centre, Royal Free London NHS Foundation Trust, London, UK; b School of Medicine, University of Leeds, Leeds, UK; c Department of Surgical Biotechnology, Division of Surgery & Interventional Science, Faculty of Medical Sciences, University College London, London, UK; d Department of Clinical Radiology, Royal Free London NHS Foundation Trust, London, UK; e Department of Interventional Radiology, Royal Free London NHS Foundation Trust, London, UK; f Department of Pharmacy, Royal Free London NHS Foundation Trust, London, UK

**Keywords:** Complex vascular malformation, mTOR inhibitors, Sirolimus, Vascular malformations

## Abstract

**Background:**

The evidence on the efficacy and safety of sirolimus therapy in patients with low-flow vascular malformations (LFVMs) has indicated its potential benefit in extensive and complicated lesions. This study aimed to assess the efficacy and safety of oral sirolimus therapy on complex LFVM patients when standard treatment alone was inadequate.

**Methods:**

This was a retrospective study of all adult patients with diagnosed LFVMs who were treated with oral sirolimus where standard therapy was inadequate in a single specialist center from May 1, 2016, to April 30, 2023. Demographic and clinical data including patient reported responses, visual analogue scores for pain and adverse effects, and quality of life (QoL) scores (Short Form-36) were reviewed.

**Results:**

We included 55 LFVM patients (14 with syndromic disease and 41 with nonsyndromic) with a median age of 41 years (range, 23-72 years). While on sirolimus, 32 patients (58.2%) experienced some improvement with a nonsignificant higher percentage of nonsyndromic patients experiencing some improvements (*P* = .6478). There was a nonsignificant improvement in the quality of life scores for physical problems, energy/fatigue, and pain. There was also a nonsignificant increase in anxiety and depression scores. There was a significant decrease in the lesion size (*P* = .0004). Two patients reported a cessation of cellulitis episodes, and eight patients reported a partial or complete reduction in bleeding from their malformation or rectal bleeding. The most common side effects reported were mouth ulcers (54.5%), fatigue (29.1%), headache (25.5%), gastrointestinal problems (25.5%), and rash (12.7%); only five patients (9.1%) did not report any side effects. No significant difference was found between the side effects reported by syndromic and nonsyndromic patients.

**Conclusions:**

Oral sirolimus therapy was clinically effective and safe in patients with complex LFVMs when standard therapy alone was inadequate. Further studies with longer follow-up are needed to evaluate oral sirolimus therapy in LFVM patients.

**Article Highlights**

• **Type of Research:** Single-center retrospective case series study
• **Key Findings:** Oral sirolimus treatment of complex and refractory vascular malformations in 55 patients resulted in a significant reduction in volume of the lesion with 32 patients experiencing some improvements. The most common side effects were mouth ulcers, fatigue, headache, gastrointestinal problems, rash, and acne.
• **Take Home Message:** Oral sirolimus therapy was clinically effective and safe in patients with extensive low-flow vascular malformation when standard therapy alone was inadequate in an National Health Service specialist center. It is important that the National Health Service would consider commissioning such treatment option for patients with refractory low-flow vascular malformations when standard therapies are inadequate.

Vascular malformations are a congenital abnormality of vascular anatomy with no pathogenic cell proliferation and can result in debilitating symptoms.^1^^,^^2^ Vascular malformations can be broadly classified as high-flow or arteriovenous malformation, and low-flow involving venous, capillary and/or lymphatic vessels.^3^ Standard management options for patients with low-flow vascular malformations (LFVMs) include conservative, medical, and invasive procedures.^4^ Conservative and medical management involve reassurance and monitoring, compression hosiery, anticoagulation, and analgesia. If the symptoms are intolerable after this, invasive procedures such as embolosclerotherapy and surgery are considered.^5^ However, not all patients with symptomatic and complicated vascular malformations respond adequately to standard management options. Moreover, invasive procedures may also carry unacceptably high risks or not be possible for some vascular malformations.

Sirolimus or rapamycin is a mammalian target of rapamycin inhibitor that acts as an immunosuppressant and was originally developed for renal transplant recipeints.^6^ In vascular malformations, it was found to inhibit endothelial and vascular smooth muscle replication.^7^ The antiproliferative properties were thought to arise from inhibition of mammalian target of rapamycin in the phosphoinositide 3-kinase and vascular endothelial growth factor expression, which are known to be responsible for the pathogenesis of many LFVMs.^8^ There is increasing evidence from nonrandomized studies to support clinical effectiveness and safety of sirolimus in patients with complicated LFVMs that were refractory to standard treatment.^9^, ^10^, ^11^ However, most of these studies were small with a relatively short follow-up and included predominantly children. The aim of this study was to investigate the efficacy and safety of sirolimus in adult patients with complex LFVMs where standard treatment alone is too high risk and/or refractory.

## Methods

This is a retrospective study of all adult patients with complex LFVMs who received oral sirolimus at a single specialist center. Sirolimus was an approved medical treatment within our vascular malformation service by the local drug and therapeutics committee with regular audit to assess its clinical efficacy and safety profile; hence, no additional research ethics approval was required. All patients received verbal counselling and written patient information leaflets for sirolimus treatment. Informed consent from patients were obtained for this study.

### Patient recruitment

All adult patients (aged ≥18 years) who presented to our specialist clinic from May 1, 2016, to April 30, 2023, were assessed by a multidisciplinary team consisting of vascular surgeons, interventional radiologists, and clinical nurse specialists for eligibility of sirolimus treatment.

### Inclusion criteria

• Symptomatic and complex LFVMs confirmed on clinical assessment and radiological scans including duplex ultrasound examination, computed tomography (CT) scan and/or magnetic resonance imaging (MRI) that were deemed too high risk, contraindicated and/or refractory to standard therapies including invasive procedures such as embolosclerotherapy and surgery.
• Age 18 years of age and older.

### Exclusion criteria

• Contraindications to sirolimus.
• Central nervous system involvement.
• Any patient who did not consent to sirolimus treatment.
• Patients who had concurrent interventional treatment for vascular malformations, such as sclerotherapy and/or surgery during the sirolimus treatment period.

### Patient pathway

**Fig 1:**
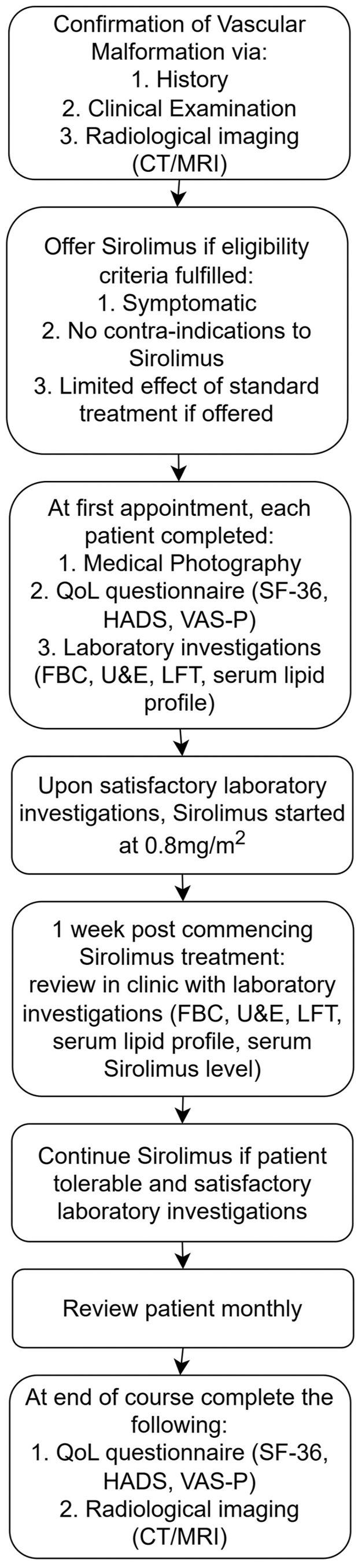
Treatment pathway of low-flow vascular malformations with sirolimus. *CT*, computed tomography; *FBC*, full blood count; *HADS*, hospital anxiety and depression score; *LFT*, liver function tests; *MRI*, magnetic resonance imaging; *QoL*, quality of life; *SF36*, 36-item short form survey; *U&E*, urea and electrolytes; *VAS-P*, visual analogue scale for pain.

Fig 1 shows the patient pathway for initiation and monitoring of sirolimus treatment for LFVM in this study.

### Pretreatment

At least 1 week before commencing sirolimus, each patient was assessed with the following baseline measures.

• Clinical history and examination including demographics, signs, and symptoms.
• Quality of Life (QoL) questionnaires: RAND 36-Item Short Form Health Survey (SF-36), Hospital Anxiety and Depression Scale (HADS), and visual analogue score for pain (VAS-P).
• Laboratory blood tests: full blood count, urea and electrolytes, liver function tests, serum lipid profile.
• MRI or CT scan of the lesion.
• Medical photography of the lesion.

### Day of commencing treatment

All patients were reviewed in the outpatient clinic, either by a face-to-face appointment or via the phone on the day sirolimus was prescribed to assess the patient at baseline and address any patient concerns. Oral sirolimus was initially prescribed at 0.8 mg/m^2^ with an aim of a plasma therapeutic range of 5 to 15 ng/mL.

### Follow-ups while taking sirolimus

Patients were followed up 1 week after commencing sirolimus treatment and then once a month thereafter.

• Clinical assessment: symptoms of their vascular malformation, side effects, and compliance.
• Laboratory blood tests: full blood count, urea and electrolytes, liver function tests, serum lipid profile, and plasma sirolimus level.
• CT scan/MRI at the end of treatment period of 6 months or more.

These measures were assessed by a clinician monthly to monitor the progression of their disease and adjust the dosage of sirolimus according to their findings.

### Stopping sirolimus

Sirolimus was discontinued if the patients were intolerant to the treatment at any time during the study period. Patients were reassessed 1 month after stopping to determine the effect of stopping sirolimus. At this appointment, the following factors was assessed.

• Clinical assessment and examination: signs and symptoms.
• QoL questionnaires: SF-36, HADS, and VAS-P.
• Medical photography of the lesion.

During the COVID-19 pandemic (started on March 23, 2020, in the UK), sirolimus therapy was stopped for most patients owing to the uncertainty of its effect on the risk of contracting and severity of COVID-19. Most patients were restarted with sirolimus after 6 to 12 months of the pandemic, but their hospital attendances and monitoring were reduced as per Public Health England's recommendations at that time. Therefore, it was not possible for some patients to complete all of the investigations and questionnaires included in the protocol. Fifteen patients completed a QoL questionnaire before and after treatment and 19 patients had an MRI before and after taking sirolimus. As phone appointments continued, self-reported symptoms by patients were well-documented in the clinic notes.

### Outcome measures

The outcome measures focus on the efficacy (clinical and radiological assessments), and safety of sirolimus at 6 months as follows.

#### Clinical assessments

• Patient-reported subjective symptom change and side effects categorized in the following categories: No improvement, mild improvement (symptoms persist that are affecting QoL without complete resolution of lesion), moderate improvement (some alleviation of symptoms without complete resolution of lesion), and significant improvement (significant improvement in symptoms and complete resolution of lesion). Any symptoms that were outlined in the initial clinic letter documenting their presenting complaint were considered whether they had improved or not. These were mainly pain, swelling, and aesthetic changes with a full list outlined in Table I.

**Table I:** Patient demographics and pretreatment clinical characteristics of all patients and by subgroups of diagnosis

Characteristics	Total	Syndromic	Nonsyndromic	*P* value
Total	55	14	41	
Age	41 [24]	44 [22]	41 [24]	.577
Sex				.702
Male	26 (47.3)	6 (42.9)	20 (48.8)	
Female	29 (52.7)	8 (57.1)	21 (51.2)	
Diagnosis				**<.001**
Nonsyndromic				
Venous malformation	35 (63.6)	0	35 (85.4)	
Capillary malformation	2 (3.6)	0	2 (4.9)	
Lymphatic malformation	4 (7.3)	0	4 (9.8)	
Syndromic				
KTS	14 (25.5)	14 (100)	0	
Anatomical location				**<.001**
Head and neck	28 (50.9)	0	28 (68.3)	
Torso	2 (3.6)	0	2 (4.9)	
Upper limb	3 (5.5)	0	3 (7.3)	
Lower limb	22 (40.0)	14 (100)	8 (19.5)	
Presenting symptom (3 most common)				
Pain	34 (61.8)	9 (64.3)	25 (61.0)	.826
Swelling	29 (52.7)	7 (50.0)	22 (53.7)	.813
Aesthetic changes	19 (34.6)	7 (50.0)	12 (29.3)	.159
Other symptoms	Discoloration, reduced range of movement, bleeding, port-wine stain, recurrent cellulitis, speech difficulties, parasthesia, dyspnea, difficulty lying flat, dysphagia, headaches, heavy limbs, trismus, vision changes, voice changes, blebs and blisters, dry skin, limb length discrepancies, menorrhagia, nodules, pitting oedema, throbbing, varicosities, visible veins			
Previous interventions				
None	9 (16.4)	1 (7.1)	8 (19.5)	.280
Surgery	28 (50.9)	10 (71.4)	18 (43.9)	.075
Embolosclerotherapy	8 (14.5)	0	8 (19.5)	.074
Sclerotherapy	24 (43.6)	4 (28.6)	20 (48.8)	.188
Laser therapy	6 (10.9)	2 (14.3)	4 (9.8)	.639
Compression hosiery	12 (21.8)	8 (57.8)	4 (9.8)	**<.001**
Embolization	3 (5.5)	1 (7.1)	2 (4.9)	.747
Medical	1 (1.8)	0	1 (2.4)	.555

*KTS,* Klippel-Trenaunay syndrome.

Values are median [interquartile range] or number (%). Boldface indicates statistical significance with a *P*-value < .05.

Syndromic = Klippel-Trenaunay syndrome.
• QoL assessment with SF-36: 36 items that measure 8 scales (physical functioning, role limitations related to physical health, bodily pain, general health, vitality, role limitations owing to emotional health, social functioning and mental health) each scored out of 100, where an increased score represents better QoL.
• HADS: assesses anxiety and depression, which comprises of seven items each to be rated on a four-point Likert scale (0 = not at all, 3 = mostly; sum score 0-21). A total score of more than 8 points denotes considerable symptoms of anxiety or depression.
• VAS-P: the score is determined by measuring on the 100 mm line between ‘no pain’ and the patient's mark, providing a range of scores from 0 to 100. A higher score indicates greater pain intensity.

#### Radiological assessment

Vascular malformation lesions were volumetrically segmented on CT scan/MRI by an independent radiologist (A.B.). Using Carestream Vue PACS (version 12.0.0.0757 lesion management tool), the vascular malformation was segmented manually by tracing each lesion with the mouse. For each patient, lesion volume was measured for before treatment and at the end of 6 months of treatment (at the end of the 6-month treatment but while still on sirolimus).

#### Safety and side effects

The safety was assessed with patient reported side effects and the laboratory test results before, during, and at the end of 6 months of therapy.

### Statistical analysis

All statistical analyses were performed using Stata version 18.0 statistical software package (Stata/MP 18.0). We performed χ^2^ tests to determine if there was a significant difference in categorical variables between syndromic and nonsyndromic patients. We used *t* tests to determine if there was a significant difference in continuous variables between syndromic and nonsyndromic patients. Wilcoxon signed-rank tests were applied to analyze for differences before and after treatment in the scores for SF-36, VAS-P, and HADS, and radiological images. *P* values of less than .05 were considered significant. Only 40 of 55 patients were included in the statistical analysis because these patients completed a full course of treatment.

## Results

### Patient demographics and pretreatment clinical characteristics

Fifty-five patients were included in this study with a median age of 41 years (range, 23-72 years). There were 26 males (47.3%) and 29 females (52.7%). The median follow-up time was 126 days (interquartile range, 424.5 days). Table I summarizes the demographics and pretreatment clinical characteristics for all patients, and within different subgroups of diagnosis of the study population. Eighty-four percent of these patients had attempted at least one intervention before starting on sirolimus.

**Fig 2:**
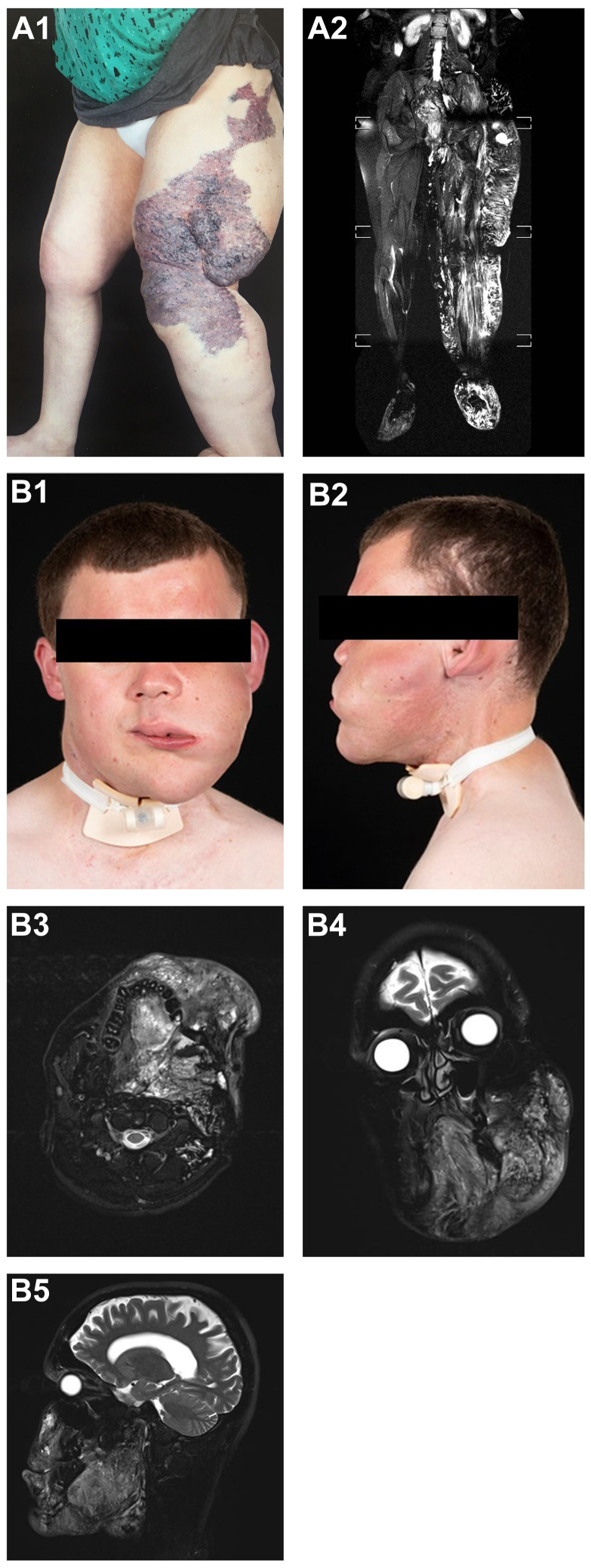
Medical photography and MRI of a 53-year-old woman lower limb Klippel-Trenaunay syndrome (KTS) **(A1-A2)** and of 23-year-old man a with facial malformation **(B1-B5)**.

All of the 14 syndromic patients had Klippel-Trenaunay syndrome (KTS) with the vascular malformations in their lower limbs. Meanwhile, 28 of the 41 nonsyndromic patients (68.3%) had vascular malformations of the head and neck. There was no statistical difference between syndromic and nonsyndromic patients for age, sex, symptoms and signs, and previous interventions (*P* > .05). The anatomical location was significantly different between syndromic and nonsyndromic patients with syndromic patients significantly more likely to have lower limb lesions. Fig 2 shows the medical photography and pretreatment MRIs for two patients.

### Patient-reported symptom changes related to the vascular malformations

Table II summarizes the patient-reported symptom changes categorized by the following categories: no improvement, mild improvement, moderate improvement, and significant improvement. Twenty-seven patients (67.5%) reported at least some degree of improvement in their condition whilst on Sirolimus. A χ^2^ test did not show a significant difference between the level of improvement in syndromic and nonsyndromic patients (χ^2^ = 3.9971; *P* = .160).

**Table II:** Frequency of improvements felt by patients taking sirolimus for patients who completed at least 6 months of treatment

Improvements	Total	Syndromic	Nonsyndromic	*P* value
No improvement	23 (41.8%)	7 (50.0)	16 (39.0%)	.5478
Mild improvement	9 (16.4%)	3 (21.4%)	6 (14.6%)	
Moderate improvement	15 (27.3%)	1 (7.1%)	14 (34.2%)	
Significant improvement	8 (14.6%)	5 (12.2%)	3 (21.4%)	

Values are number (%).

### QoL assessment

Table III summarizes the median of each domain of the SF-36 questionnaire, HADS, and VAS-P before and after treatment for the 15 patients who completed the full course of Sirolimus. The median scores for role limitations owing to physical problems, energy/fatigue, and pain increased, which were not statistically significant. However, for general health, physical functioning, emotional well-being, and social functioning the scores decreased also nonsignificantly. HADS and VAS-P scores were similar from before treatment to at the end of 6 months of treatment. When the Wilcoxon signed-rank test was applied, there was no statistically significant differences found between the pre- and 6-months treatment scores in any of the eight domains of SF-36, HADS, and VAS-P (*P* > .05).

**Table III:** Pre- and postsirolimus treatment quality of life (QoL), anxiety, depression, and pain scores

	Total (n = 15)			Syndromic (n = 2)			Nonsyndromic (n = 13)		
	Before treatment	After treatment	*P* value	Before treatment	After treatment	*P* value	Before treatment	After treatment	*P* value
SF-36 domains									
General health	60 (45)	45 (45)	.6230	27.5 (25)	27.5 (25)	>.9999	60 (25)	50 (40)	.5996
Physical functioning	70 (55)	65 (45)	.5449	25 (50)	25 (50)	>.9999	80 (50)	80 (40)	.5410
Role limitations (physical)	37.5 (100)	60 (45)	.2393	0 (0)	25 (50)	>.9999	87.5 (100)	65 (45)	.3711
Role limitations (emotional)	100 (100)	100 (67)	.6250	0 (0)	16.7 (33.3)	>.9999	100 (50)	100 (33.3)	>.9999
Energy/fatigue	35 (55)	45 (30)	.1860	27.5 (15)	35 (0)	>.9999	60 (55)	45 (30)	.1860
Emotional well-being	72 (36)	70 (40)	.5508	70 (4)	72 (0)	>.9999	72 (36)	68 (40)	.5371
Social functioning	75 (62.5)	62.5 (62.5)	.7227	25 (0)	25 (0)	>.9999	75 (62.5)	62.5 (50)	.7188
Pain	57.5 (55)	67.5 (45)	.1953	56.3 (2.5)	55 (0)	>.9999	57.5 (55)	67.5 (42.5)	.1797
HADS									
Anxiety	4 (6)	7 (7)	.5918	4.2 (3.7)	7.2 (9.7)	>.9999	4 (6)	7 (5)	.8257
Depression	3 (8)	5 (6)	.9163	6.5 (7)	4 (2)	>.9999	3 (4.8)	5 (6)	.5708
Pain									
VAS-P	25 (68)	25 (45)	.7935	47.5 (45)	40 (60)	>.9999	20 (58)	25 (40)	0.9595

*HADS,* Hospital anxiety and depression scale; *IQR,* interquartile range; *VAS-P,* visual analogue score for pain.

Values are number (%).

### Pre- and postsirolimus treatment radiological assessment

**Fig 3:**
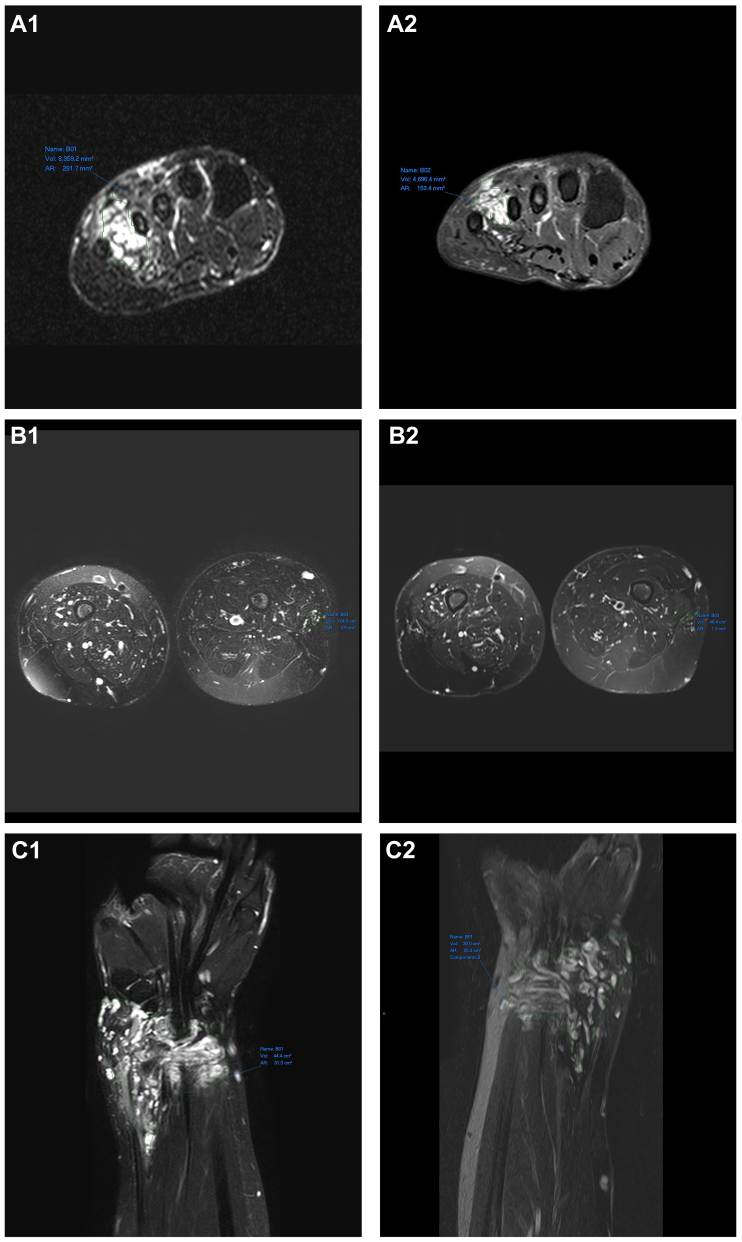
Magnetic resonance imaging (MRI) before treatment **(A1-C1)** and after treatment **(A2-C2)** with sirolimus. The lesion volume decreased in these three patients.

Table IV shows the volume of the VM lesion for 19 of the patients who completed a course of sirolimus and had an available MRI before and after. For 17 of these 19 patients, the volume of their lesion decreased in size. Fig 3 shows the MRIs of three participants. The median volume of the pretreatment lesions was 51.6 cm^3^ and 40.3 cm^3^ for the post-treatment lesions. The median percentage change was 20.0%. A Wilcoxon signed-rank test showed a statistically significant reduction in lesion size (*z* = 3.380; *P* = .0002). For patients with a syndromic malformation, the mean size of the volume of the lesion decreased, but not statistically significantly (*z* = 1.604; *P* = .2500). However, there were only three syndromic patients and 16 nonsyndromic patients for whom a full set of MRIs were available. For patients with a nonsyndromic malformation, a Wilcoxon signed-rank test showed a statistically significant reduction in volume of the lesion (*z* = 2.999; *P* = .0013).

**Table IV:** Pre- and postsirolimus treatment vascular malformation lesion size, measured in area, on magnetic resonance imaging (MRI)

Lesion size in volume before treatment, cm^3^	Lesion size in volume after treatment, cm^3^	% Change
50.6	44.1	Reduced by 12.8%
18.6	15.9	Reduced by 14.5%
59.8	40.2	Reduced by 32.8%
23.5	18.8	Reduced by 20.0%
40.5	33.2	Reduced by 18.0%
44.4	30.0	Reduced by 32.4%
23.0	29.6	28.7% increase
73.4	64.0	Reduced by 12.8%
104.5	46.4	Reduced by 55.6%
38.9	32.6	Reduced by 16.2%
145.5	88.0	Reduced by 39.5%
62.5	40.3	Reduced by 35.5%
95.6	60.4	Reduced by 36.8%
36.4	38.8	6.6% increase
81.0	75.0	Reduced by 7.4%
135.5	129.8	Reduced by 4.2%
52.6	40.8	Reduced by 22.4%
17.9	12.7	Reduced by 29.1%
8.4	4.7	Reduced by 44.0%

### Infection

Seven patients had recurrent cellulitis and lesion infection before starting sirolimus treatments, six of whom had syndromic disease; one had nonsyndromic disease. Of these patients, only five completed a full course of sirolimus and, of those, two reported a cessation of cellulitis episodes.

### Coagulopathy

At baseline, nine patients experienced bleeding from their LFVM (six with venous malformations, two with lymphatic malformations, and one with KTS) and three experienced rectal bleeding (one with a pelvic and rectal venous malformation and two with left leg KTS). Rectal bleeding was defined as any self-reported bleeding into the toilet, on tissue, or into the underwear from rectum. Bleeding from the LFVM was reported as any fresh, red blood from the LFVM site. One patient with bleeding from a facial lymphatic malformation stopped sirolimus after 2 months owing to fatigue and cold-like symptoms. Four of the remaining patients with bleeding from their malformation reported a partial or complete decrease in bleeding from their malformation. A patient with rectal bleeding and left lower limb KTS stopped taking sirolimus after 1 month owing to no improvement and intolerable side effects. The two remaining patients with rectal bleeding reported an improvement in bleeding symptoms.

### Safety and side effects

Six patients (42.9%) with syndromic LFVM could not tolerate sirolimus. Table V shows the six most frequent side effects reported by the patients. The most common side effect reported was mouth ulcers (54.5%). None of these effects showed a statistically significant difference between the syndromic and nonsyndromic patients. In those patients who stopped sirolimus therapy, their side effects resolved on cessation of the medication. No patient who was on sirolimus had a significant problem related to COVID-19 infection during the pandemic.

**Table V:** Most common reported side effects during the treatment period for total patients and separated into syndromic and nonsyndromic

Side effect	All patients	Syndromic	Nonsyndromic	*P* value
None	5 (9.1)	0	5 (12.2)	.171
Mouth ulcers	30 (54.5)	5 (35.7)	25 (61.0)	.101
Fatigue	16 (29.1)	4 (28.6)	12 (29.3)	.960
Headache	14 (25.5)	5 (35.7)	9 (22.0)	.307
GI-related side effects	14 (25.5)	3 (21.4)	11 (27.8)	.689
Rash	7 (12.7)	2 (14.3)	5 (12.2)	.839
Acne	6 (10.9)	0	6 (14.6)	.129

*GI,* Gastrointestinal.

Six venous malformation patients and two KTS patients reported an infection while taking sirolimus. One patient experienced a fungal skin infection. One patient reported that they were more susceptible to coughs and colds since taking sirolimus, but also reported that they worked with children. Another patient experienced systemic symptoms of an infection while on sirolimus, but no source of the infection was found.

Since starting sirolimus, three patients stopped a course of sirolimus owing to contracting a chest infection, two of whom had LFVM and one had KTS. One of these patients resumed sirolimus without reporting any further issues with infection.

## Discussion

The understanding of the pathogenesis of vascular malformations has evolved over the years, with increased revelation of identified gene association, has enabled a role for targeted medical therapy. Medical therapy is useful in treating patients where standard treatment alone is challenging, too high risk, and/or refractory.

Our study has shown that 67.5% of patients reported some improvement in symptoms whilst taking sirolimus. In addition, there was a decrease in the volume of vascular malformations after a minimum of a 6-month course of sirolimus. Because 29 patients (52.7%) reported swelling and 15 (27.3%) reported aesthetic concerns as symptoms of their vascular malformation, a decrease in size could improve the health of patients with LFVMs significantly. There was a nonsignificant improvement in the QoL scores for role limitations owing to physical problems, energy/fatigue, and pain. The median VAS-P scores did not increase. The most common side effects experienced by patients were mouth ulcers, fatigue, headache, gastrointestinal discomfort, rash, and acne (Table V).

Sirolimus was shown to decrease the volume of LVFM lesions in patients with extensive disease and are being treated at a tertiary center, but can have significant, sometimes debilitating side effects. The change in QoL and pain while on sirolimus were difficult to determine. The amount patients are willing to tolerate their side effects is subjective and must be a personal decision made by the patient with the aid of their clinician. For the patients for whom the benefits outweigh the effect of the side effects, sirolimus can improve their health and well-being.

Sirolimus has been used for managing vascular malformations since 2010. A systematic review of 73 studies found the most common side effects to be oral mucositis (32%), gastrointestinal symptoms (10%), and rash (8%), which was similar to our study, but at a lower incidence.^12^ This study included many diagnoses of varying severity, whereas the patients in our study have some of the most severe vascular malformations in the UK. Additionally, there were no KTS patients in the systematic review, but KTS makes up a quarter of our patients. Another potential source of difference in the incidence of side effects is the younger age of the patients in the systematic review (19 months) compared with 41 years in this study.

Another recent study found that sirolimus significantly decreased the lesion volume of head and neck LFVMs.^11^ The preliminary results of a phase III trial of LFVM patients observed a clinical benefit rate of 85% with improvements within the first month experienced by 50% of patients.^13^ Our study has demonstrated sirolimus therapy was safe and effective in patients with extensive disease when standard treatment alone was challenging, too high risk and/or refractory. Our findings were comparable with existing literature, but we have also demonstrated reduction in lesion size from MRI. We have also compared the effectiveness of sirolimus in syndromic patients (KTS), but further investigation with a larger sample size is needed to make definitive conclusions.

A retrospective review of patients in a single center over 9 years found that 92% of patients had partial response or stable disease while on sirolimus and describes sirolimus as well-tolerated.^14^ Whereas our study of more severe cases of VM showed fewer patients experienced improvements while on sirolimus.

Two small studies of pediatric vascular malformation patients found similar results to our study. A study of 25 pediatric patients in a Beijing hospital found pain relief within 2 months and to be effective in venous malformation patients with seven of eight venous malformation patients experiencing improvements.^15^ Another study of pediatric vascular anomaly patients taking sirolimus showed that the disease did not worsen and was safe and well-tolerated, but did not include information of the change in size of the lesion.^10^ Our study found that sirolimus was also safe and effective in some adult patients and significantly reduced the size of the lesion.

A limitation of this study was the amount of missing data for MRI results and QoL scores because these forms were mostly completed on paper when patients attended the clinic in person, which were changed to phone appointments during the COVID-19 pandemic. Many patients were also advised to stop sirolimus therapy in the first 6 months of the COVID-19 pandemic, but restarted it subsequently. It was not until 2023 when an online form was used. Therefore, many patients did not fill out the SF-36 questionnaire before undergoing sirolimus treatment. Additionally, many MRI scans were also cancelled to reduce the risk patients on sirolimus from contracting infections while attending hospital as they were immunocompromised. Therefore, a further study with multiple methods of collecting questionnaire data from patients to accommodate the change from in person to online clinics would provide more data on how QoL changes while taking sirolimus.

A second limitation of this study was the small sample size, decreasing reliability. Therefore, a larger study including more patients would provide more conclusive evidence on the effect of sirolimus on the QoL of LFVM patients. A larger study would enable stratification of the data to determine if sirolimus is more effective in different types of lesions or different anatomical locations. For example, stratification may provide deeper insight into which patients sirolimus was effective for.

Another limitation of this study was the lack of a control group to compare against the experience of VM patients without sirolimus treatment. This meant that we cannot draw conclusions on the effect of sirolimus compared with other treatments or monitoring. Furthermore, the participants were not randomized, so the decision to start sirolimus was influenced by selection bias.

## Conclusions

Oral sirolimus seems to be safe and effective in treating some patients with complex LFVMs who are too high risk and/or refractory to standard treatment alone. This modality provides an additional option for patients with intolerable symptoms and complications from LFVM, for whom more invasive interventions are too high risk or have been ineffective. Further studies are needed to further quantify this and determine the long-term effects of sirolimus.

## Author contributions

Conception and design: RN, CP, NE, MK, AP, JB, CSL

Analysis and interpretation: RN, CP, CSL

Data collection: RN, CP, NE, AB, MK, AP, TR, JB, CSL

Writing the article: RN, CP

Critical revision of the article: RN, CP, NE, AB, MK, AP, TR, JB, CSL

Final approval of the article: RN, CP, NE, AB, MK, AP, TR, JB, CSL

Statistical analysis: RN

Obtained funding: CSL

Overall responsibility: CSL

RN and CP contributed equally to this article and share co-first authorship.

## Funding

C.P. received funding from the Royal Free Charity, London, UK. This study was supported by researchers (C.S.L.) at the 10.13039/501100000272National Institute for Health Research 10.13039/501100012621University College London Hospitals Biomedical Research Centre. The Royal Free Vascular Anomalies also received funding from the Butterfly AVM Charity.

## Disclosures

None.
